# Multiscale understanding of tricalcium silicate hydration reactions

**DOI:** 10.1038/s41598-018-26943-y

**Published:** 2018-06-04

**Authors:** Ana Cuesta, Jesus D. Zea-Garcia, Diana Londono-Zuluaga, Angeles G. De la Torre, Isabel Santacruz, Oriol Vallcorba, Monica Dapiaggi, Susana G. Sanfélix, Miguel A. G. Aranda

**Affiliations:** 1 ALBA Synchrotron, Carrer de la Llum 2-26. 08290 Cerdanyola del Vallès, Barcelona, Spain; 2 Departamento de Química Inorgánica, Cristalografía y Mineralogía. Universidad de Málaga, 29071 Málaga, Spain; 3 Department of Earth Sciences “Ardito Desio”, University of Milan, Milano, Italy; 4 Faculty of Engineering, Østfold University College, N-1757 Halden, Norway

## Abstract

Tricalcium silicate, the main constituent of Portland cement, hydrates to produce crystalline calcium hydroxide and calcium-silicate-hydrates (C-S-H) nanocrystalline gel. This hydration reaction is poorly understood at the nanoscale. The understanding of atomic arrangement in nanocrystalline phases is intrinsically complicated and this challenge is exacerbated by the presence of additional crystalline phase(s). Here, we use calorimetry and synchrotron X-ray powder diffraction to quantitatively follow tricalcium silicate hydration process: i) its dissolution, ii) portlandite crystallization and iii) C-S-H gel precipitation. Chiefly, synchrotron pair distribution function (PDF) allows to identify a defective clinotobermorite, Ca_11_Si_9_O_28_(OH)_2_^.^8.5H_2_O, as the nanocrystalline component of C-S-H. Furthermore, PDF analysis also indicates that C-S-H gel contains monolayer calcium hydroxide which is stretched as recently predicted by first principles calculations. These outcomes, plus additional laboratory characterization, yielded a multiscale picture for C-S-H nanocomposite gel which explains the observed densities and Ca/Si atomic ratios at the nano- and meso- scales.

## Introduction

Le Châtelier^1^ already established that Portland cement hydration starts by the dissolution of calcium silicate species in water from the most soluble silicate phase. This process is followed by the precipitation of complex poorly-crystalline calcium-silicate-hydrates (generically named C-S-H gel) and the crystallization of Ca(OH)_2_, portlandite, see overall reaction (1)^2^. C-S-H gel is the main hydrated component in Portland cement pastes, and it is the main responsible for the strength and durability of the resulting mortars and concretes. Alite, an impure form of tricalcium silicate Ca_3_SiO_5_, is the main phase present in Portland cements and it has a slightly variable composition due to element-substitutions^2^. The hydration of any alite shows, in addition to an initial fast (minor) dissolution, three main stages with time: i) induction (also known as dormant period), ii) acceleration; and iii) deceleration^3^. Similar kinetic profiles take place in various heterogeneous hydration processes, for instance mineral weathering^4^ and glass alteration^5^. There are two main theories to explain this early-age hydration behaviour. The first is known as ‘protective layer’ and it consists in the precipitation of a C-S-H gel diffusion barrier on the surfaces of alite particles which density and adherence change with time. The second is known as ‘geochemical model’ and it is related to the alite dissolution mechanism evolving from etch pit formation to step retreat^6^. Despite one century of focused investigations, the underlying mechanism(s) for such time evolution is still strongly debated^7,8^.

1 $${{\rm{Ca}}}_{{\rm{3}}}{{\rm{SiO}}}_{{\rm{5}}}+5{{\rm{.2H}}}_{{\rm{2}}}{\rm{O}}\to {\rm{1}}{\rm{.2Ca}}{({\rm{OH}})}_{{\rm{2}}}+{({\rm{CaO}})}_{{\rm{1.8}}}{{\rm{SiO}}}_{{\rm{2}}}{({{\rm{H}}}_{{\rm{2}}}{\rm{O}})}_{{\rm{4.0}}}$$

The hydration reactions of alite, (i) dissolution of crystalline alite, (ii) precipitation of C-S-H gel, and (iii) crystallization of portlandite, have been thoroughly studied by many techniques including laboratory X-ray powder diffraction^9–12^, calorimetry^13–15^, small-angle neutron scattering^16^; advanced electron microscopies^17,18^, ^29^Si magic-angle-spinning nuclear-magnetic-resonance^19,20^, and theoretical simulations^21–23^. C-S-H gel has a nanocrystalline nature and so its understanding is very challenging^24^ which includes the relationship with the solution where it is equilibrated^25^. There are many reviews addressing this component and we cite just the most relevant and recent ones^26–28^. An updated mechanism of growth of C-S-H gel has been very recently proposed^29^. It is also important to add that the hydration of alite is affected by the presence of other species. This has been very recently exemplified by the study of the influence of aluminates, added as NaAlO_2_, on the hydration kinetics of alite which was studied by a multi technique approach including molecular dynamics simulations to investigate the dissolution step at the nanoscale^30^.

Many studies have shown that the C-S-H gel aggregates contain poorly-crystalline interconnected nanoparticles described as globules, disks and foils^31–36^ which enclose water within nanopores, known as gel pore water^26^. This gel water can evolve with time with consequences in C-S-H ‘bulk’ density^37^. It is worth noting that this water within the gel is different from the capillary pore water (also known as free water, FW) as it cannot be removed without altering the properties of the system^27^. Concerning the atomic arrangement within the nanocrystalline component of the C-S-H gel aggregates, several experimental and theoretical techniques have concluded that defective clinotobermorite is the best available approximation^37–40^. Crystalline tobermorite-14Å, Ca_5_Si_6_O_16_(OH)_2_.7H_2_O, has a Ca/Si ratio and density of 0.83 and 2.19 gcm^−3^, respectively. The corresponding values for crystalline tobermorite-11Å, Ca_4_Si_6_O_15_(OH)_2_.5H_2_O, are 0.67 and 2.40 gcm^−3^, respectively^41^. These previous observations are striking as they are not in straightforward agreement with two well-established key bulk measured properties: i) the Ca/Si ratio on the C-S-H aggregates ranges between 1.6–2.0; and ii) the density of nanoglobules (gel pore water excluded) range 2.5–2.6 g·cm^−3 ^^2,31,37^. Defective clinotobermorite could justify a Ca/Si atomic ratio close to 1.2–1.3, but not higher than that^42^. Very fine intermixing of C-S-H gel with calcium hydroxide has been proposed from electron microscopy^43,44^ which it could explain an overall Ca/Si ratio ranging 1.6–2.0.

**Figure 1:**
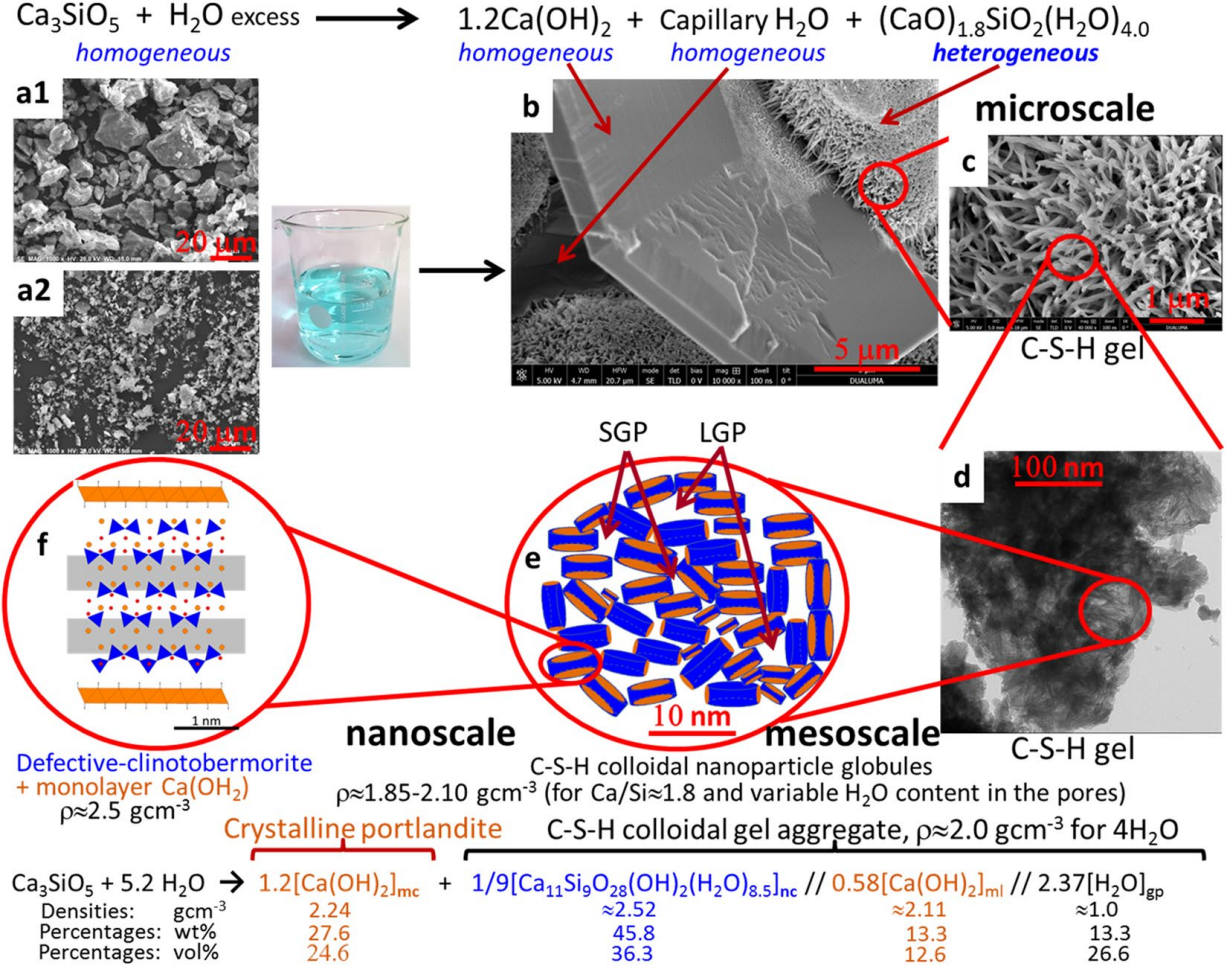
Schematic understanding of the alite hydration reaction at different length scales. (Top) Hydration reaction of tricalcium silicate at the microscale. (**a1**) SEM microphotograph for C_3_S_21 µm. (**a2**) SEM microphotograph for C_3_S_3 µm. (**b**) SEM microphotograph for C_3_S_21 µm_080 paste showing a homogeneous portlandite plate microparticle, voids arising from capillary water, and three agglomerates of heterogeneous C-S-H gel. (**c**) Enlarged view of one C-S-H gel region in (**b**). (d) TEM microphotograph of C_3_S_3 µm_080_arrested:16d showing interspersed foil-like C-S-H nanoparticles at the mesoscale. (**e**) Schematic representation of the C-S-H colloidal nanoparticles of clinotobermorite (blue) and monolayer Ca(OH)_2_ (orange) generating the small gel pores (SGP) and large gel pores (LGP) of Jennings’s model (25). (**f**) Schematic representation of a single C-S-H nanoglobule composed by defective clinotobermorite and two monolayers of Ca(OH)_2_ at the nanoscale. (Bottom) Hydration reaction of tricalcium silicate at the nanoscale highlighting the three main components of colloidal C-S-H nanocomposite: nanocrystalline clinotobermorite, amorphous (monolayer) calcium hydroxide and gel pore water. The (approximate) densities, mass and volume percentages of the different components are also given for an overall water content of four water molecules per silicate.

The aim of this work is to contribute to the understanding of both the formation and nanostructure of C-S-H gel. Firstly, we used synchrotron X-ray powder diffraction (and calorimetry) for *in situ* determining the dissolution of alite as well as the crystallization of portlandite and the precipitation of the gel. Secondly, we have employed synchrotron X-ray total scattering (and ^29^Si MAS-NMR) to study the short- and medium- range atomic arrangement in the C-S-H gel nanoparticles. With this knowledge and observations from electron microscopy and previous reports, we propose a model for this complex heterogeneous system, developing a multiscale picture (see Fig. 1) for the hydration of alite in order to explain the observed mass densities and Ca/Si atomic ratios at the different scales. At the nanoscale, below 10 nm, C-S-H gel are composed of a fine intermixing of defective clinotobermorite, particle sizes ranging 3–5 nm with Ca/Si ratio close 1.2, and monolayers of Ca(OH)_2_. These aggregates generate the gel pores. At the mesoscale, between 10 and 100 nm, neat C-S-H gel appears relatively heterogeneous with (CaO)_1.8_SiO_2_(H_2_O)_4.0_ overall composition but slightly variable Ca/Si atomic ratios in different volumes. This is now explained by slightly different defective clinotobermorite to Ca(OH)_2_-monolayers local ratios. At the microscale, above 100 nm, the hydration reaction of alite is well known resulting in crystalline Ca(OH)_2_, also named portlandite, C-S-H gel and capillary water, see Fig. 1.

## Results

### *In situ* calorimetric study

**Figure 2:**
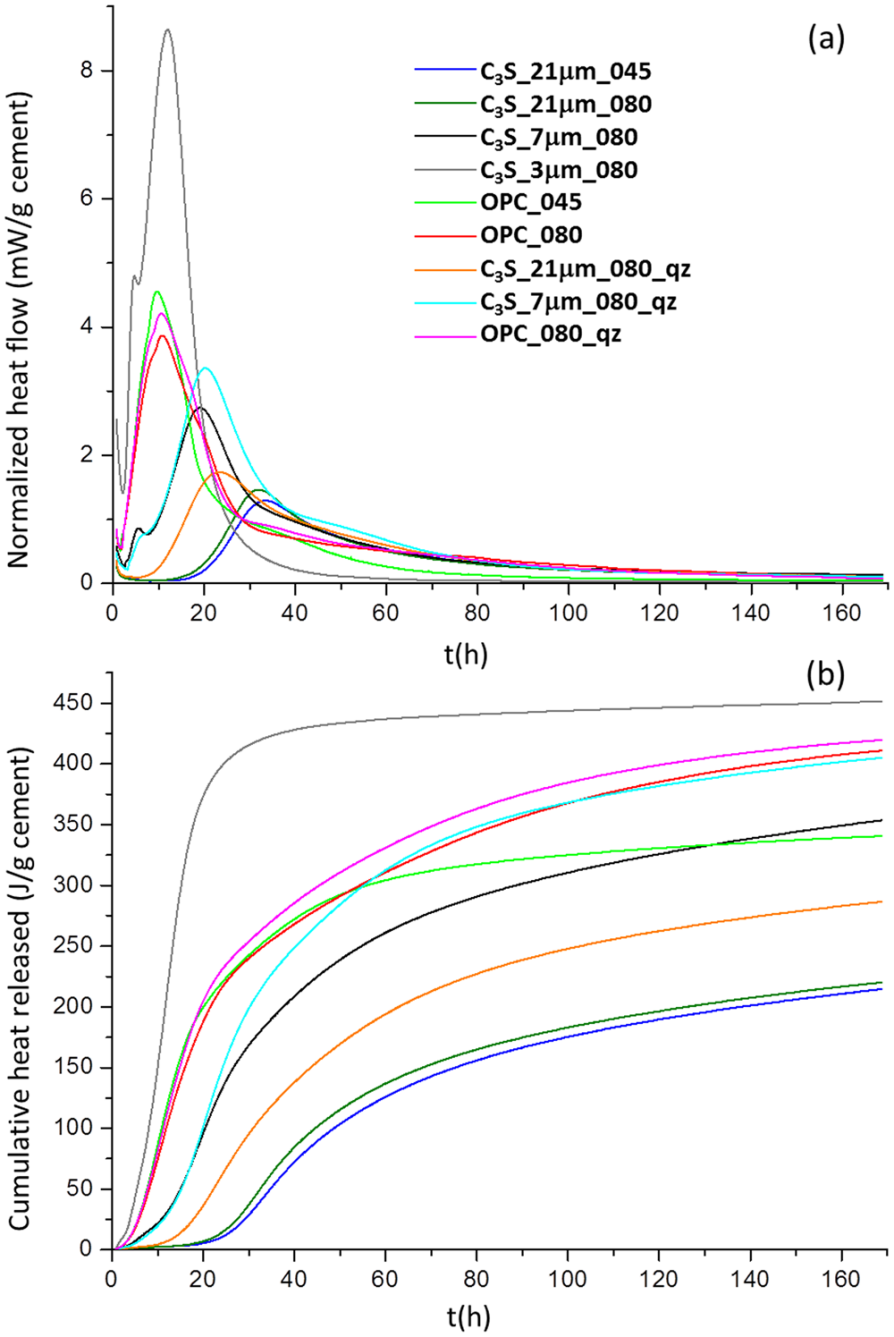
Calorimetric data. (**a**) Heat flow calorimetry curves and (**b**) Cumulative heat released for C3S_21 µm_045, C3S_21 µm_080, C3S_7 µm_080, C3S_3 µm_080, C3S_21 µm_080_qz, and C3S_7 µm_080_qz. Data collected in the same run for a Portland cement type-I (OPC_045 and OPC_080) and OPC_080_qz are given as reference.

The hydration reactions of tricalcium silicate were studied *in situ* by calorimetry and synchrotron X-ray powder diffraction (SXRPD). The particle size distribution (PSD) (diameter) for as-received alite was quite large, D_v,50_ = 20.8 µm, see Supplementary Fig. 1a. Therefore, two additional samples were prepared, see methods, with D_v,50_ = 7.4 and 2.7 µm, Supplementary Fig. 1b,c, respectively. The values of D_v,10_ and D_v,90_ are also shown in Supplementary Fig. 1. The characterization of these three alites is given in the supplementary information. Heat flow calorimetry curves and cumulative heat released traces up to seven days are shown in Fig. 2a,b, respectively. Table 1 reports the key values obtained from the calorimetric study including the alite reaction degree that can be estimated as the overall heat of hydration for tricalcium silicate is known^2^, 517 Jg^−1^. This is an approximation as the impurities in alite can play a role as well as the structural defects. The four studied pastes are labelled as C_3_S_21 µm_045, C_3_S_21 µm_080, C_3_S_7 µm_080 and C_3_S_3 µm_080 to highlight their PSDs and water-to-alite mass ratios, i.e. 045 means a ratio of 0.45. C_3_S_21 µm_080_qz and C_3_S_21 µm_080_qz stands for samples with 10 wt% of quartz and their calorimetries were recorded for the sake of comparison with the SXRPD study where 10 wt% of quartz as internal standard was employed.

**Table 1:** Key values obtained from the alite calorimetries.

Sample	BET surface area (m^2^/g)	t (h)^1^	Heat-1 (J/g cement)^2^	α-1 (%)^3^	Heat-2 (J/g cement)^4^	α-2 (%)^5^
C_3_S_21 µm_045	0.3(1)	33	43.2	8.4	214.2	41.4
C_3_S_21 µm_080	0.3(1)	32	46.1	8.9	219.9	42.5
C_3_S_21 µm_080_qz	—	23	55.4	10.7	286.3	55.4
C_3_S_7 µm_080	1.1(1)	19	88.4	17.1	353.2	68.3
C_3_S_7 µm_080_qz	—	20	106.1	20.5	404.9	78.3
C_3_S_3 µm_080	5.2(1)	12	209.1	40.4	451.2	87.3

^1^Time at the maximum of the heat flow curves. ^2^Total heat evolved at the maximum of heat flow curves. ^3^Reaction degree at the maximum of heat flow curves. ^4^Total heat evolved at seven days. ^5^Reaction degree at seven days.

### *In situ* synchrotron X-ray powder diffraction study

SXRPD data taken in capillaries of unaltered pastes were analyzed by Rietveld methodology employing the internal standard method, in this case quartz, for amorphous quantification^45^. For this *in situ* study, the overall amorphous values encompass not only the C-S-H gel content but also the FW because the hydration reactions were not arrested. To a first approximation, the bound water (the crystallization water and the gel pore water) can be calculated according to reaction (1), as the amount of dissolved alite is known. Then, this calculated value is subtracted from the initial amount of water, in order to obtain the amount of FW for each hydration age. Finally, all the weight percentages were recalculated excluding the amount of FW in order to follow the evolution of the C-S-H gel component, which it includes the nanopore gel water and any amorphous calcium hydroxide.

**Figure 3:**
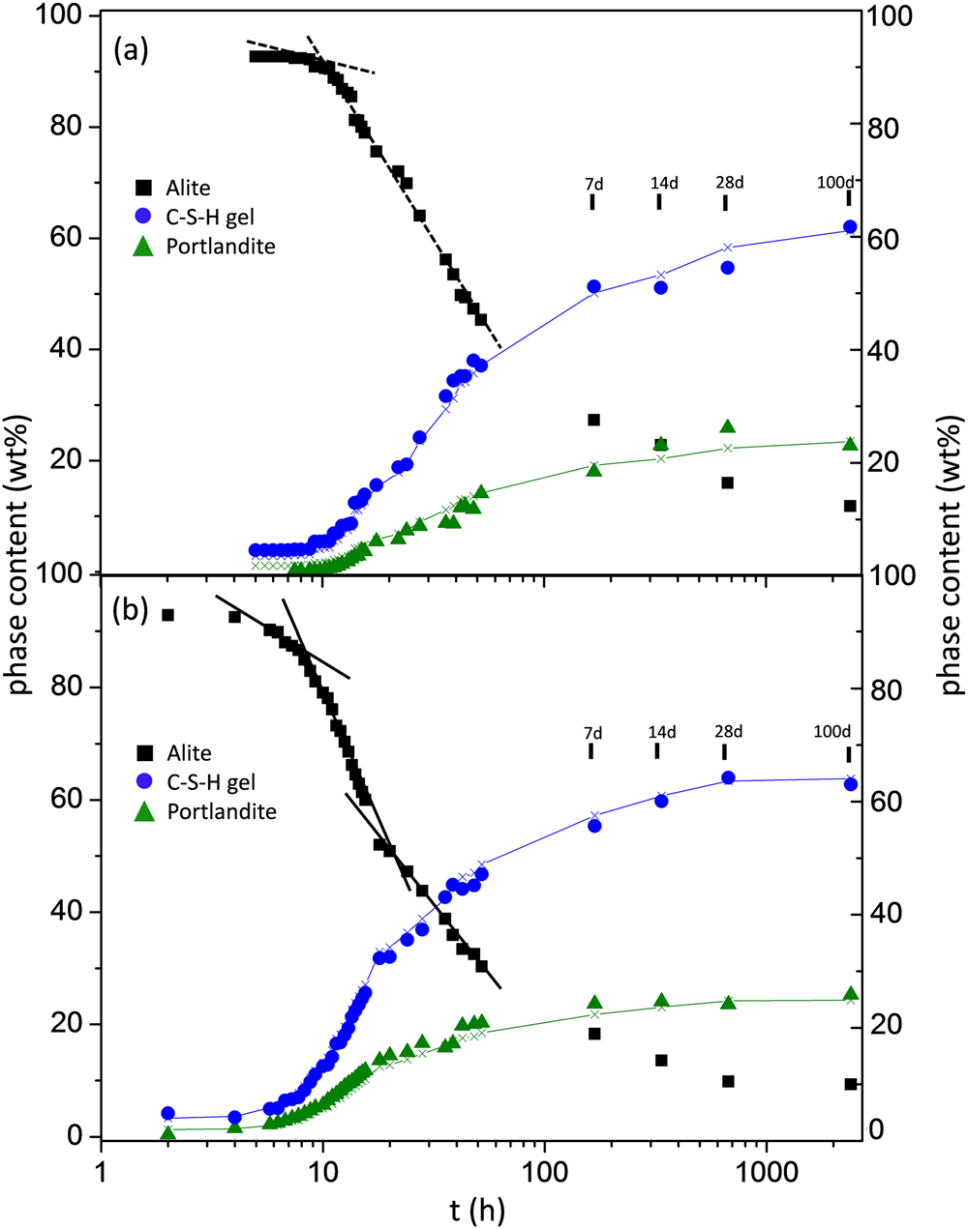
Quantitative phase analysis results from SXRPD. (**a**) C_3_S_21 µm_080 and (**b**) C_3_S_7 µm_080. The lines for C-S-H gel (blue) and portlandite (green) show the theoretical amounts expected from the measured dissolution of alite according to reaction (1).

Figure 3a shows the phase content evolution with time for C_3_S_21 µm_080 paste from SXRPD data. It is worth noting that, at 5 hours of hydration (which was our first measurement), alite was partly dissolved, ≈4 wt%, and ≈4 wt% of C-S-H gel had precipitated. The crystallization of portlandite started later, close to 7.5 hours. At 14 hours, only 2.8 wt% of crystalline portlandite was measured. The hydration reaction progressed slowly up to 100 days of hydration as it can be shown in Fig. 3a. As an example, Supplementary Fig. 2a shows the Rietveld plot for this paste at 14 hours of hydration. Finally, the quantitative phase analysis results obtained at 100 days (2400 h) was 11.8, 22.7 and 62.1 wt% of unreacted alite, crystalline portlandite and C-S-H gel, respectively. The remaining content, 3.4 wt%, was belite, Ca_2_SiO_4_.

Figure 3b shows a similar study for C_3_S_7 µm_080. For this sample, the hydration kinetics is faster. At the first measurement time, 2 hours, 4.6 wt% of alite was already dissolved, with the precipitation of ≈4 wt% of C-S-H gel and the crystallization of ≈0.4 wt% of portlandite. At 14 hours, 10.4 wt% of crystalline portlandite was measured. Supplementary Fig. 2b shows the Rietveld plot for this paste at 14 hours of hydration. A transition from the accelerated hydration reaction kinetics to decelerated kinetics is clearly observed in the phase content evolutions close to 20 hours of hydration. At 100 days of hydration, 9.3 wt% of unreacted alite was measured coexisting with 25.3 wt% of portlandite and 62.8 wt% of C-S-H gel. The remaining content, 2.6 wt% was belite, Ca_2_SiO_4_.

### ^29^Si MAS-NMR and electron microscopy studies

**Figure 4:**
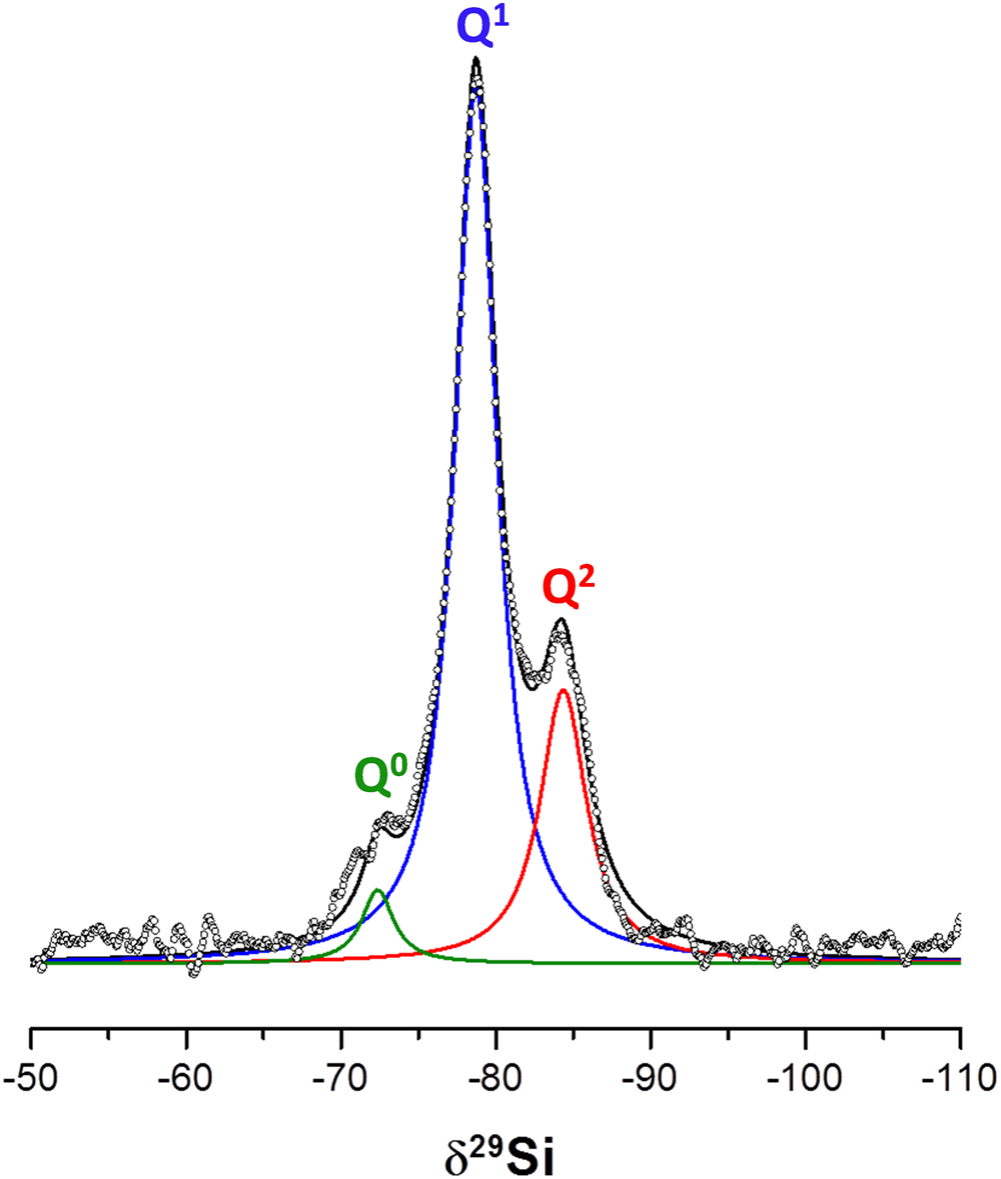
^29^Si MAS-NMR spectra for C3S_3 µm_080_arrested:16d measured. Spinning rate of 15 kHz and a magnetic field of 14.1 T.

The alite sample with the smallest PSD, see Supplementary Fig. 1c, was hydrated in order to minimize the amount of unreacted alite. The hydration was arrested, see methods section, in order to remove the FW, and this paste is labelled hereafter C_3_S_3 μm_080_arrested:16d. Rietveld quantitative phase analysis of LXRPD after 16 days of hydration, see Supplementary Fig. 3a, gave: 1.1 wt% of unreacted alite, 20.5 wt% of crystalline portlandite, 2.5 wt% of crystalline calcium carbonate and 75.9 wt% of amorphous content (mainly but not necessarily only C-S-H gel). Supplementary Fig. 3b shows the simulated X-ray diffraction pattern for the defective clinotobermorite T3_14sc structure, average particle size ≈5 nm, which has been used in the PDF study to fit the contribution of the nanocrystalline C-S-H gel. C_3_S_3 μm_080_arrested:16 d was also studied by ^29^Si MAS-NMR, see Fig. 4, compared to related samples in Supplementary Fig. 4, and electron microscopy, see Supplementary Figs 5 and 6. ^29^Si MAS-NMR data give direct information about the silicate chains in C-S-H gel. The signals observed at −78.7 and −84.4 ppm were attributed to the Q^1^ and Q^2^ Si units, respectively^46,47^. Q^1^ is associated with silicate end chain units and Q^2^ indicates the presence of silicate in intermediate chain positions^48,49^. The very weak signal at −72.4 ppm corresponds to isolated Q^0^ tetrahedra from unreacted alite. The silicate MCL (mean chain length) can be determined from the expression^48^, MCL = 2(Q^1^ + Q^2^)/Q^1^. The deconvolution of the spectrum shown in Fig. 4 gave 70.8% for Q^1^ and 24.7% for Q^2^. Therefore, MCL was 2.70. This value agreed well with previous reports for early age pastes^48,49^. The ^29^Si MAS-NMR spectra for two related samples gave very similar MCL values, see Supplementary Fig. 4. In addition, the determination of the average Ca/Si ratio is also an important parameter in any investigation of a C-S-H gel. Supplementary Fig. 5 shows a HRTEM micrograph with EDS data as an example. From 64 analysed points, the average Ca/Si atomic ratio was 1.75 ± 0.16. Supplementary Fig. 6 displays a FEGSEM micrograph (fracture cross-section).

### Total-scattering pair distribution function study

**Figure 5:**
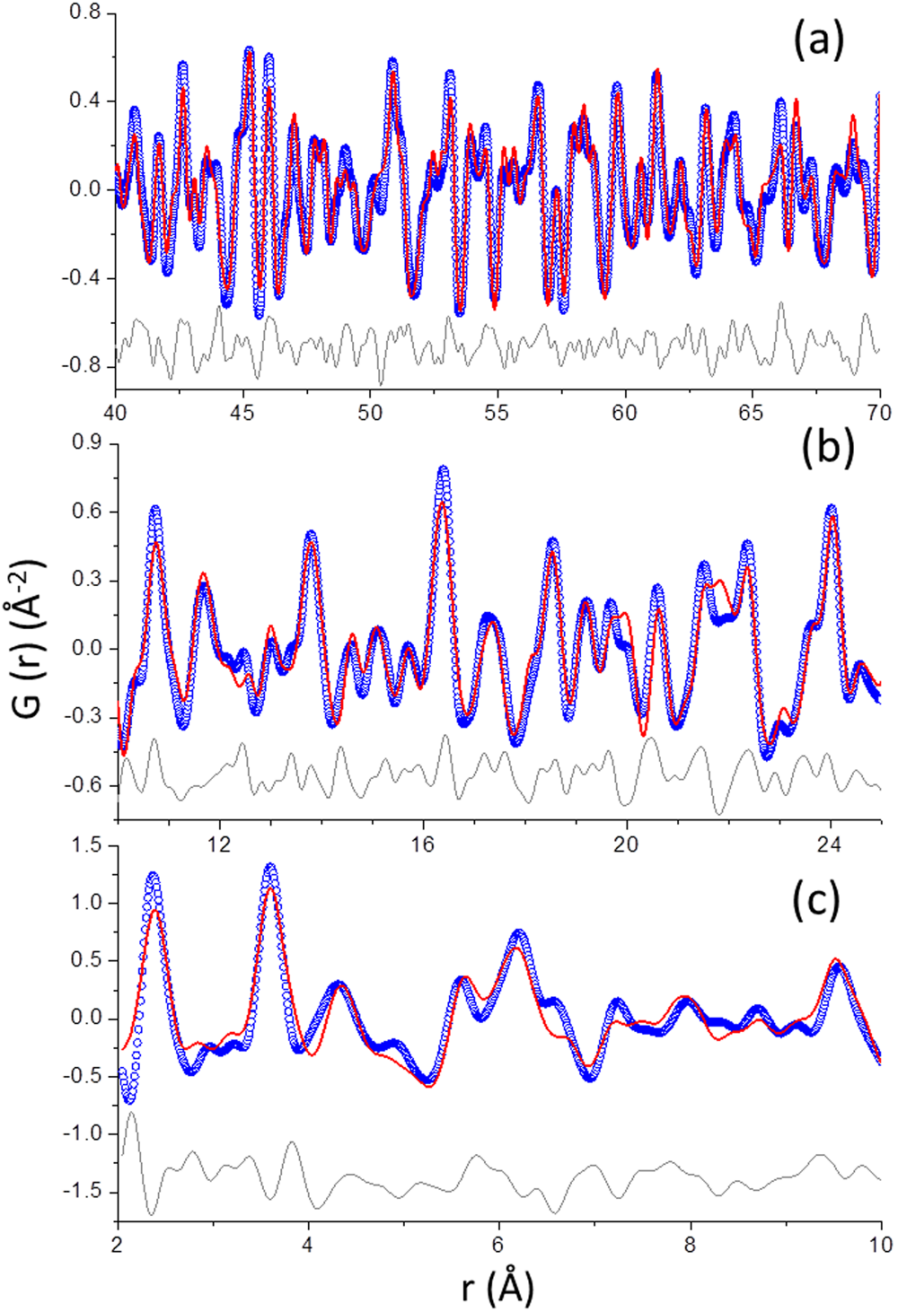
PDF refinements. Experimental (blue circles), fitted (red lines) and difference (grey lines) PDF patterns for C_3_S_3 µm_080_arrested:16d (**a**) from 40 to 70 Å; (**b**) from 10 to 25 Å; and (**c**) from 2 to 10 Å. For details of the fits, the readers are referred to the text.

The PDF data for C_3_S_3 μm_080_arrested:16d, see Fig. 5, have been analyzed using the same strategy previously reported by us^40^. Initially, a high r-region, i.e. 40–70 Å, was analyzed with the contributions of the crystalline phases: portlandite and alite. The low amount of alite was computed as discussed in the supplementary information. The calcite content converged to zero. After refining all parameters, the final R_W_ value was 26.9%. The fit is displayed in Fig. 5a. The unit cell values for portlandite converged to a = 3.595 Å and c = 4.918 Å. Secondly, all the previously determined parameters were kept fixed and the contribution from the nanocrystalline component of C-S-H gel was investigated in the r-region, from 10 to 25 Å. As it was previously reported^40^, several crystal structures have been tested (Hillebrandite, Jennite, stoichiometric Tobermorite-14, stoichiometric Tobermorites-11 (monoclinic and orthorhombic), stoichiometric clinotobermorites, and selected structural descriptions from ref.^42^ which had a MCL close to 3.0) to fit the contribution of the nanocrystalline C-S-H gel, see Supplementary Table 1. Jennite structure led to the worst PDF fit as evidenced by its higher R_W_ value, see Supplementary Fig. 7. Hillebrandite has now been included as it has been very recently reported as a good model for the nanocrystalline component of C-S-H gel^50^ but it gave a poor fit to our PDF data. Supplementary Table 1 gives the R_W_ values for each PDF fit and the quantitative phase analysis results. The defective clinotobermorite structure, T3_14sc^42^ with Ca_11_Si_9_O_28_(OH)_2_·8.5H_2_O composition, has been selected as it gave the best fit to the C-S-H gel nanoparticle contribution to the PDF profile. The unit cell values for the defective clinotobermorite T3_14sc structure converged to a = 11.255 Å, b = 7.320 Å, c = 42.415 Å and β = 94.2° and the isotropic ADPs were 0.0083 and 0.0160 Å^2^ for Ca, Si, respectively. The R_W_ was 27.7% and the final fit is displayed in Fig. 5b. For all the PDF fits, the ADP (U-thermal displacement parameter) value for O was not refined, the value was fixed to 0.070 Å^2^ with correspond to that of as received Ca_3_SiO_5_. The PDF fit gave the following quantitative phase analysis results: 2.2 wt% of anhydrous alite, 33.5 wt% of portlandite and 64.3 wt% of clinotobermorite.

Thirdly, the low r-region, 2 to 10 Å, was studied. The PDF fit based on the contributions of portlandite, alite and defective clinotobermorite resulted in a difference PDF curve, which was quite large, see Supplementary Fig. 8, especially in the very low r-region, suggesting an amorphous component. The scattering misfit closely corresponds to the theoretical PDF trace for an isolated monolayer of Ca(OH)_2_ as recently suggested^39,40^. Therefore, we have included a monolayer of Ca(OH)_2_, atoms arranged as in portlandite, in the PDF fits, see supplementary information. The fit of the difference curve indicates that this monolayer Ca(OH)_2_ is expanded along *a* and *c* directions, 3.8 and 5.3%, respectively and compressed along *b* direction, 2.1%. Overall, the volume of monolayer Ca(OH)_2_ is expanded 7.0% when compared to the volume of crystalline portlandite. This volume expansion would lead to a density decrease from 2.24 gcm^−3^ in bulk portlandite to 2.11 gcm^−3^ in monolayer portlandite. The stretched monolayer portlandite structure was then used in the PDF refinement (Fig. 5c). As expected, the larger correlation in this refinement took place between the scale factors of monolayer-Ca(OH)_2_ and crystalline-Ca(OH)_2_, −55%. The Rw value for the final fit, without computing alite, see Fig. 5c, dropped from 57 to 38% due to the contribution of monolayer portlandite.

## Discussion

The hydration kinetic of as received alite was slow with the maximum in the heat flow curve close to 30 hours, with a reaction degree of ∼9%, see Table 1 and Fig. 2. The presence of 10 wt% of crystalline quartz accelerates the hydration and the maximum takes place at 23 hours, with a reaction degree of 10.7% for the paste hydrated at a w/s = 0.80. This slow kinetics was due to the large average particle size, ≈20 μm, and its relatively high iron content, 1.1 wt% expressed as Fe_2_O_3_. It is known that iron^51^ and chromium^52^ strongly delay alite hydration. Conversely, its kinetic is only slightly dependent on the water-to-alite ratio as previously reported^14,53^. The calorimetric and SXRPD studies showed a strong acceleration of alite hydration for smaller particle size samples, see Table 1 and Figs 2 and 3, in agreement with previous reports^11,54^. It is worth noting that the small peak evident in the heat flow curve at 5 h for C_3_S_7 µm_080, see Fig. 2, is very likely due to its bimodal PSD with a minimal fraction of very small particle size close to 0.6 μm, see Supplementary Fig. 1b. The acceleration due to the presence of quartz, known as ‘filler effect’^55^, is much more pronounced for the alite sample with the largest particles. This observation point towards that alite dissolution is kinetically the limiting reaction.

We underline that the first 45 minutes of hydration is not recorded in our calorimetric study and this could neglect the measurement of the hydration of ≈5% of alite, according to the results from the SXRPD study. In any case is worth comparing the reaction degrees from calorimetry (with quartz!), Table 1, and those obtained from the quantitative analysis of SXRPD data, Fig. 3. For C_3_S_21 µm_080_qz at 23 h (the time of the maximum of the heat flow curve), a reaction degree (as defined by the transformed fraction) of 11% (it could be ≈16% taking into account the fast dissolution) is obtained from the calorimetric study and ≈19% is obtained from the synchrotron study. For C_3_S_7 µm_080_qz at 20 h, a reaction degree of 21% (which it could be ≈26%) is obtained from the calorimetric study and ≈38% is obtained from the synchrotron study. We justify this disagreement due to the main differences between the two set of experiments: i) the rotation of the capillary in the diffraction study with its associated shear effect;^15^ and ii) and the slightly higher temperature of the diffraction study when compared to the calorimetric study, see methods. It is key to compare results from different techniques but it is also important to understand the role of the different experimental conditions that sometimes are necessary to ensure the maximum attainable accuracy.

It has been reported from *in situ* laboratory powder diffraction data, Bragg-Brentano geometry, that the crystalline portlandite content was about one third smaller than that expected from alite dissolution according to reaction (1)^10,56^. This is not the case in our synchrotron powder diffraction study as the used methodology (rotating the capillary in transmission and merging data from three capillary positions), improves the accuracy of the analyses. Figure 3 also displays the expected amounts of portlandite and C-S-H gel from alite dissolution which were in very good agreement with the measured values. Finally, it is worth noting that at very early hydration ages, the measured crystalline portlandite contents are slightly smaller than the expected ones. We speculate that this could be due to the initial precipitation of amorphous calcium hydroxide.

At 100 days, the RQPA results for C_3_S_21 µm_080 were 11.8, 22.7 and 62.1 wt% of unreacted alite, crystalline portlandite and C-S-H gel, respectively. The corresponding values for C_3_S_7 µm_080 were 9.3, 25.3 and 62.8 wt%, respectively. According to equation (1), the expected amounts of portlandite and C-S-H gel were 23.4 and 61.4 wt%, and 24.4 and 63.7 wt%, for C_3_S_21 µm_080 and C_3_S_7 µm_080, respectively. The good agreement between the determined and expected contents, for both samples, indicates the accuracy of the methodology and the suitability of the stoichiometric coefficients in reaction (1).

The PDF analysis of the total scattering data for C_3_S_3 μm_080_arrested:16d paste agrees well with our previous report^40^. Moreover, the very small amount of unreacted alite, lower than 2 wt%, yielded a PDF study with less uncertainties. The crystal structure which gave the best fit to the PDF data in the region 10 to 25 Å was invariably clinotobermorite, see Supplementary Table 1. We have selected defective clinotobermorite T3_14sc, Ca_11_Si_9_O_28_(OH)_2_·8.5H_2_O, because in addition to the very good fit this approximate structure represents a ‘trimer’ derived from a staggered-chain clinotobermorite^42^. More studies are needed to confirm if this structural description is the best option for the hydration of alite under different conditions and in Portland cements. Its average silicate chain length of 3.0 is in agreement with the MCL value, 2.7, obtained by ^29^Si MAS-NMR. However, its Ca/Si ratio, 1.22, does not agree with the Ca/Si ratio of the C-S-H determined by electron microscopy, 1.75, which is widely reported in bibliography^2,44^ and previously confirmed by reaction (1). However, these Ca/Si ratios are not obtained at the same probing length scales. The Ca/Si ratio of defective clinotobermorite measured by PDF analysis is obtained at the nanoscale, probing scale: 1–3 nm. The Ca/Si ratio measured by HAADF-STEM is obtained at the mesoscale, probing scale ≈50–100 nm. The Ca/Si ratios determined from FEGSEM and Rietveld refinement of SXRPD data are obtained at the microscale, probing scale >500 nm. The apparent disagreement in the Ca/Si ratios can be reconciliated by the hypothesis that C-S-H gel aggregates arising from the hydration of alite, is a composite formed by a fine intermixing at the nanoscale of defective clinotobermorite nanoglobules, sizes ≈4 nm, and amorphous Ca(OH)_2_, size <2 nm, see Fig. 1.

The overall multiscale picture for the hydration of alite is shown in Fig. 1 where reaction (1) at the microscale is depicted on top and its breakdown at the nanoscale is depicted at the bottom. The densities of the different components are also reported as well as their percentages (mass and volume). The hypothesis of existence of monolayer Ca(OH)_2_ and its fine intermixing with defective clinotobermorite is supported in three ways. I. The PDF analysis in the 2–10 Å region improves notably with the inclusion of monolayer portlandite, R_W_ decreases from 57 to 38%. Unfortunately, the analysis of the scales factors does not allow to obtain the weight percentage of the monolayer Ca(OH)_2_ component. II. The determined expansion of monolayer Ca(OH)_2_ in this study, 7.0%, is in agreement with the expansion recently predicted by first principles calculations when studying pure calcium hydroxide systems^57^. This density functional theory theoretical work reported the stability of monolayer Ca(OH)_2_ and an expansion of the average Ca-O bond from 2.36 to 2.38 Å from crystalline portlandite to monolayer Ca(OH)_2_. III. The PDF analysis in the 10–25 Å length scale gave crystalline portlandite and nanocrystalline clinotobermorite contents of 33.5 and 64.3 wt%, respectively (see Supplementary Table 1). Our model reported at the bottom of Fig. 1, and rescaled to take into account 2 wt% of unreacted alite, gives 36.8 and 61.1 wt%, respectively. The relative close agreement between these two sets of values can be also interpreted as an indirect support of the model.

## Conclusion

The *in situ* SXRPD study has confirmed the stoichiometry of alite hydration reaction to yield portlandite and C-S-H gel with (CaO)_1.8_SiO_2_(H_2_O)_4.0_ average composition. Chiefly, by using high-resolution synchrotron PDF analysis, it has been found that C-S-H gel is heterogeneous at the nanoscale being composed of defective clinotobermorite, with approximate composition Ca_11_Si_9_O_28_(OH)_2_·8.5H_2_O, and monolayers of Ca(OH)_2_. With these results and observations from electron microscopy and previous reports, a multiscale model for the hydration of alite is proposed (see Fig. 1) which explains the observed mass densities and Ca/Si atomic ratios at the relevant scales. At the nanoscale, below 10 nm, C-S-H gel are composed of a fine intermixing of defective clinotobermorite, particle sizes ranging 3–5 nm with Ca/Si ratio close 1.2 and ρ≈2.5 gcm^−3^, and monolayers of Ca(OH)_2_, ρ≈2.1 gcm^−3^. The calcium silicate component justifies the previously reported nanoglobules density, ρ≈2.6 gcm^−3^^31,37^. These aggregates generate the gel pores. At the mesoscale, between 10 and 100 nm, neat C-S-H gel appears with variable compositions, Si/Ca ratio and water content, centred at (CaO)_1.8_SiO_2_(H_2_O)_4.0_. This is explained by slightly different defective clinotobermorite to Ca(OH)_2_-monolayers local ratios and the variable gel pore water. Uneven water content also justifies the observed C-S-H gel densities at this scale, 1.9–2.1 gcm^−3 ^^26^ At the microscale, above 100 nm, heterogeneous (CaO)_1.8_SiO_2_(H_2_O)_4.0_ gel and homogeneous portlandite are arranged enclosing volumes of water, termed capillary water. The picture reported here should be taken into account for developing theoretical models. Furthermore, synthetic C-S-H gels (with Ca/Si ratios <1.4) may have very different properties as the monolayer calcium hydroxide component could be absent. Finally, the new model explains a striking feature of the hydration of cements blended with fly ash where portlandite content is measured to decrease much less than predicted by thermodynamic modelling. Although portlandite is still present, the Ca/Si ratio in C-S-H is observed to decrease from close to 1.8 in plain pastes to close to 1.4 in fly ash blends^58^. This is now explained by the consumption of Ca(OH)_2_ monolayer component of the C-S-H gel in the pozzolanic reaction.

## Methods

Full details about the Methods can be found in the supplementary information

### Sample preparation

Monoclinic tricalcium silicate, alite, was acquired from Mineral Research Processing M.R.PRO. Its chemical composition determined by XRF was: 72.3 wt% CaO, 25.5 wt% SiO_2_, 1.1 wt% Fe_2_O_3_, 0.5 wt% MgO and 0.5 wt% Al_2_O_3_. For the *in situ* synchrotron X-ray powder diffraction studies, the anhydrous mixtures were mixed with 10.00 wt% of SiO_2_ (99.5%, AlfaAesar) as an internal standard^45^.

C_3_S_21 µm_080 labels the paste produced by using as received alite with a water-to-alite mass ratio of 0.80. C_3_S_7 µm_080: The as received alite was milled in a vibratory mill (Retsch, mod. MM200) and the resulting powder was mixed with a water-to-alite mass ratio of 0.80. For the PDF study, as received alite was attrition milled and hydrated at a water-to-solid mass ratio of 0.80 for 16 days at 20 °C. Finally, the hydration stoppage procedure was performed by solvent exchange with isopropanol and ether^59^. This sample was labelled C_3_S_3 μm_080_arrested:16d.

For the sake of comparison in the calorimetry studies, an Ordinary Portland Cement (OPC) from Financiera y Minera S.A. has been used. The samples have been labelled as OPC_045, OPC_080 and OPC_080_qz to indicate the w/s mass ratios. Furthermore, label _qz denotes the OPC with added quartz as standard.

### Particle Size Distribution (PSD)

Average particle size and particle size distribution for the alite samples were measured using a laser analyzer, Mastersizer S, Malvern, UK.

### BET surface area

The specific surface areas of the selected samples were measured by multi-point N_2_ adsorption with a BET (ASAP 2420, Micromeritics, USA) instrument.

### Calorimetry

The isothermal calorimetric study was performed in an eight channel Thermal Activity Monitor (TAM) instrument using glass ampoules. The heat flow was collected up to 7 days at 20 °C.

### Thermal analysis

Differential thermal analysis (DTA) and thermogravimetric (TGA) measurement for C_3_S_3 μm_080_arrested:16d was performed in a SDT-Q600 analyzer from TA instruments (New Castle, DE).

### NMR study

^29^Si MAS-NMR (Magic Angle Spinning Nuclear Magnetic Resonance) spectrum for C_3_S_3 μm_080_arrested:16d was recorded at RT on a Bruker AVIII HD 600 NMR spectrometer (field strength of 14.1 T) at 156.4 MHz. The Chemical shift was referenced to an external solution of tetramethylsilane.

### Laboratory X-ray powder diffraction (LXRPD) with internal standard

LXRPD data for C_3_S_3 μm_080_arrested:16d was collected on a D8 ADVANCE (Bruker AXS) diffractometer (SCAI – Universidad de Malaga) equipped with a Johansson monochromator, using strictly monochromatic Mo-Kα_1_ radiation, λ = 0.7093 Å, in transmission geometry (θ/θ). Sample was mixed with 20 wt% of internal standard (α-Al_2_O_3_).

### Synchrotron X-ray powder diffraction (SXRPD)

For the phase evolution study, SXRPD patterns were collected in Debye-Scherrer (transmission) mode using the X-ray powder diffraction endstation of BL04-MSPD beamline at ALBA synchrotron (Barcelona, Spain). The total acquisition time was 6 min per dataset. For the PDF study, SXRPD data for C_3_S_3 μm_080_arrested:16d were collected for 3 h at the same diffractometer. The temperature inside the experimental hutch was 28 °C.

### Rietveld data analysis

Rietveld analyses were performed using the GSAS suite of programs and the EXPGUI graphic interface^60^. The non-crystalline content (amorphous and nanocrystalline) was determined by the internal standard methodology^45^.

### Pair Distribution Function data analysis

PDF experimental data were obtained using PDFgetX3^61^ with Q_max_ = 21 Å^−1^. Quantitative phase analysis was obtained by using the PDFgui software^62^ and CMI-diffpy complex modeling software^63^.

### Electron microscopy study

High resolution transmission electron microscopy (HRTEM) measurements were carried out using a FEI Talos F200X microscope equipped with X FEG and super-X EDS (Energy Dispersive Spectroscopy) system with four silicon drift detectors (SDDs) which operates at an accelerating voltage of 200 kV. FEGSEM (Field Emission Gun Scanning Electron Microscopy) micrographs and EDS analysis were performed in a Helios Nanolab 650 Microscope (FEI Company) with a retractable CBS Backscatter detector (annular solid-state device) and X-Max 50 mm^2^ detector (Oxford instruments).

### Data Availability

All synchrotron X-ray powder diffraction raw data files underlying this article can be accessed on Zenodo at 10.5281/zenodo.1027759, and used under the Creative Commons Attribution license.

## Electronic supplementary material

Supplementary information

## References

[1] Le Chatelier, H. *Recherches Expérimentales sur la Constitution des Mortiers Hydrauliques* (Doctoral thesis, Faculté des Sciences de Paris, 1887).

[2] Taylor, H. F. W. *Cement Chemistry* (Thomas Telford, London, 1997)

[3] Bullard JW (2011). Mechanisms of Cement Hydration. Cem. Concr. Res..

[4] Nugent MA, Brantley SL, Pantano CG (1998). & Maurice, P. A. The influence of natural mineral coatings on feldspar weathering. Nature.

[5] Cailleteau C (2008). Insight into silicate-glass corrosion mechanisms. Nat. Mater..

[6] Scrivener KL, Juilland P, Monteiro PJM (2015). Advances in understanding hydration of Portland cement. Cem. Concr. Res..

[7] Nicoleau L, Nonat A, Daval D (2018). Rate-limiting reaction of C_3_S hydration - A reply to the discussion “A new view on the kinetics of tricalcium silicate hydration” by E. Gartner. Cem. Concr. Res..

[8] Gartner E (2018). Discussion of the paper “A new view on the kinetics of tricalcium silicate hydration,” by L. Nicoleau and A. Nonat, Cem. Concr. Res. 86 (2016) 1–11. Cem. Concr. Res..

[9] Jansen D, Bergold ST, Goetz-Neunhoeffer F, Neubauer J (2011). The hydration of alite: a time-resolved quantitative X-ray diffraction approach using the G-factor method compared with heat release. J. Appl. Crystallogr..

[10] Bergold ST, Goetz-Neunhoeffer F, Neubauer J (2013). Quantitative analysis of C-S-H in hydration alite pastes by *in-situ* XRD. Cem. Concr. Res..

[11] Bergold ST, Goetz-Neunhoeffer F, Neubauer J (2015). Mechanically activated alite: New insights into alite hydration. Cem. Concr. Res..

[12] Bergold ST, Goetz-Neunhoeffer F, Neubauer J (2016). Influence of the reactivity of the amorphous part of mechanically activated alite on its hydration kinetics. Cem. Concr. Res..

[13] Lerch W, Bogue RH (1934). Heat of hydration of Portland cement pastes. J. Res. Natl. Bur. Stand..

[14] Thomas JJ (2007). A New Approach to Modeling the Nucleation and Growth Kinetics of Tricalcium Silicate Hydration. J. Am. Ceram. Soc..

[15] Juilland P, Kumar A, Gallucci E, Flatt RJ (2012). & Scrivener K. L. Effect of mixing on the early hydration of alite and OPC systems. Cem. Concr. Res..

[16] Thomas JJ, Allen AJ, Jennings HM (2009). Hydration Kinetics and Microstructure Development of Normal and CaCl_2_-Accelerated Tricalcium Silicate Pastes. J. Phys. Chem. C.

[17] Kjellsena KO, Lagerblad B (2007). Microstructure of tricalcium silicate and Portland cement systems at middle periods of hydration-development of Hadley grains. Cem. Concr. Res..

[18] Wenzel O (2017). Investigating the pore structure of the calcium silicate hydrate phase. Mater. Character..

[19] Bellmann F, Damidot D, Möser B, Skibsted J (2010). Improved evidence for the existence of an intermediate phase during hydration of tricalcium silicate. Cem. Concr. Res..

[20] Pustovgar E (2016). Understanding silicate hydration from quantitative analyses of hydrating tricalcium silicates. Nat. Comm..

[21] Abdolhosseini-Qomi MJ (2014). Combinatorial molecular optimization of cement hydrates. Nat. Comm..

[22] Ioannidou K (2016). Mesoscale texture of cement hydrates. Proc. Natl. Acad. Sci. USA.

[23] Ioannidou K (2016). The crucial effect of early-stage gelation on the mechanical properties of cement hydrates. Nat. Comm..

[24] Thomas JJ, Jenning HM, Allen AJ (2010). Relationships between composition and density of tobermorite, jennite, and nanoscale CaO−SiO_2_−H_2_O. J. Phys. Chem. C.

[25] Lothenbach B, Nonat A (2015). Calcium silicate hydrates: solid and liquid phase composition. Cem. Concr. Res..

[26] Jennings HM (2008). Refinements to colloid model of C-S-H in cement: CM-II. Cem. Concr. Res..

[27] Papatzani S, Paine K, Calabria-Holley J (2015). A comprehensive review of the models on the nanostructure of calcium silicate hydrates. Constr. Build. Mater..

[28] Palkovic SD (2016). Roadmap across the mesoscale for durable and sustainable cement paste–A bioinspired approach. Constr. Build. Mater..

[29] Gartner E, Maruyama I, Chen J (2017). A new model for the C-S-H phase formed during the hydration of Portland cements. Cem. Concr. Res..

[30] Pustovgar E (2017). Influence of aluminates on the hydration kinetics of tricalcium silicate. Cem. Concr. Res..

[31] Allen AJ, Thomas JJ, Jennings HM (2007). Composition and density of nanoscale calcium-silicate-hydrate in cement. Nat. Mater..

[32] Masoero E, Del Gado E, Pellenq RJ-M, Ulm F-J, Yip S (2012). Nanostructure and Nanomechanics of Cement: Polydisperse Colloidal Packing. Phys. Rev. Lett..

[33] Chiang W-S (2013). Microstructural changes of globules in calcium–silicate–hydrate gels with and without additives determined by small-angle neutron and X-ray scattering. J. Colloid. Interface Sci..

[34] Cappelletto E (2013). Comb-Shaped Polymers as Nanostructure Modifiers of Calcium Silicate Hydrate: A ^29^Si Solid-State NMR Investigation. J. Phys. Chem. C.

[35] Chiang W-S (2014). Multiscale structure of calcium- and magnesium-silicate-hydrate gels. J. Mater. Chem. A.

[36] Yu Z, Zhou A, Lau D (2016). Mesoscopic packing of disk-like building blocks in calcium silicate hydrate. Sci. Rep..

[37] Muller ACA, Scrivener KL, Gajewicz AM, McDonald PJ (2013). Densification of C–S–H Measured by 1H NMR Relaxometry. J. Phys. Chem. C.

[38] Rejmak P, Dolado JS, Stott MJ, Ayuela A (2013). ^29^Si Chemical Shift Anisotropies in Hydrated Calcium Silicates: A Computational Study. J. Phys. Chem. C.

[39] Grangeon S (2017). Quantitative X-ray pair distribution function analysis of nanocrystalline calcium silicate hydrates: a contribution to the understanding of cement chemistry. J. Appl. Crystallogr..

[40] Cuesta A (2017). Synchrotron radiation pair distribution function analysis of gels in cements. Crystals.

[41] Dharmawardhana CC, Misra A, Ching W-Y (2014). Quantum Mechanical Metric for Internal Cohesion in Cement Crystals. Sci. Rep..

[42] Richardson IG (2014). Model structures for C-(A)-S-H(I). Acta. Crystallogr. B.

[43] Thomas JJ, Chen JJ, Jennings HM, Neumann DA (2003). Ca−OH bonding in the C−S−H gel phase of tricalcium silicate and white Portland cement pastes measured by inelastic neutron scattering. Chem. Mater..

[44] Chen JJ, Sorelli L, Vandamme M, Ulm FJ, Chanvillard GJ (2010). A coupled nanoindentation/SEM-EDS study on low water/cement ratio portland cement paste: evidence for C–S–H/Ca(OH)2 nanocomposites. J. Am. Ceram. Soc..

[45] Aranda MAG, De la Torre AG, León-Reina L (2012). Rietveld quantitative phase analysis of OPC clinkers, cements and hydration products. Rev. Mineral. Geochem..

[46] Goñi S (2010). Quantitative study of hydration of C_3_S and C_2_S by thermal Analysis. Evolution and composition of C–S–H gels formed. J. Therm. Anal. Calorim..

[47] Mendes A, Gates WP, Sanjayan JG, Collins F (2011). NMR, XRD, IR and synchrotron NEXAFS. Mater. Struct..

[48] Richardson IG (1999). The nature of C-S-H in hardened cements. Cem. Concr. Res..

[49] Sanchez-Herrero MJ, Fernandez-Jimenez A, Palomo A (2016). Alkaline hydration of C_2_S and C_3_S. J. Am. Ceram. Soc..

[50] Bae S (2017). Pair distribution function analysis of nanostructural deformation of calcium silicate hydrate under compressive stress. J. Am. Ceram. Soc..

[51] Stephan D, Wistuba S (2006). Crystal structure refinement and hydration behaviour of 3CaO·SiO_2_ solid solutions with MgO, Al_2_O_3_ and Fe_2_O_3_. J. Eur. Ceram. Soc..

[52] Lua L, Xianga C, He Y, Wang F, Hu S (2017). Early hydration of C_3_S in the presence of Cd^2+^, Pb^2+^ and Cr^3+^ and the immobilization of heavy metals in pastes. Constr. Build. Mater..

[53] Kirby DM, Biernacki JJ (2012). The Effect of Water-to-Cement Ratio on the Hydration Kinetics of Tricalcium Silicate Cements: Testing the Two- Step Hydration Hypothesis. Cem. Concr. Res..

[54] Masoero E, Thomas JJ, Jennings HM (2014). A reaction zone hypothesis for the effects of particle size and water-to-cement ratio on the early hydration kinetics of C_3_S. J. Am. Ceram. Soc..

[55] Berodier E (2014). & Scrivener, K. Understanding the Filler Effect on the Nucleation and Growth of C-S-H. J. Am. Ceram. Soc..

[56] Bergold ST, Goetz-Neunhoeffer F, Neubauer J (2017). Interaction of silicate and aluminate reaction in a synthetic cement system: Implications for the process of alite hydration. Cem. Concr. Res..

[57] Aierken Y (2015). Portlandite crystal: Bulk, bilayer, and monolayer structures. Phys. Rev. B.

[58] Lothenbach, B. & Winnefeld, F. Thermodynamic modelling of cement hydration: Portland cements – blended cements – calcium sulfoaluminate cements in *Cementitious Materials - Composition, Properties, Application* (ed Pollmann, H.) 103–143 (Walter de Gruyter, Berlin, 2017).

[59] García-Mate MDla, Torre AG, León-Reina L, Aranda MAG, Santacruz I (2013). Hydration studies of calcium sulfoaluminate cements blended with fly ash. Cem. Concr. Res..

[60] Larson, A. C. & Von Dreele, R. B. General Structure Analysis System (GSAS). *Los Alamos National Laboratory Report LAUR*, pp 86–748 (2000).

[61] Juhàs P, Davis T, Farrow CL, Billing SJL (2013). PDFgetX3: a rapid and highly automatable program for processing powder diffraction data into total scattering pair distribution functions. J. Appl. Crystallogr..

[62] Farrow CL (2007). PDFfit2 and PDFgui: computer programs for studying nanostructure in crystals. J. Phys. Condens. Matter..

[63] Juhás P, Farrow CL, Yang X, Knox KR, Billinge SJL (2015). Complex modeling: a strategy and software program for combining multiple information sources to solve ill posed structure and nanostructure inverse problems. Acta. Crystallogr. A.

