# Supplementary material for: Multiscale understanding of tricalcium silicate hydration reactions
Source: Sci Rep. 2018 Jun 4;8:8544. doi: 10.1038/s41598-018-26943-y (PMC5986785; doi:10.1038/s41598-018-26943-y)
Supplement: Supplementary file 1 — Supplementary information [file 41598_2018_26943_MOESM1_ESM.docx]

Supplementary information submitted to *Scientific Reports*

Multiscale understanding of the tricalcium silicate hydration reactions

Ana Cuesta^a,b^, Jesus D. Zea-Garcia^b^, Diana Londono-Zuluaga^b^, Angeles G. De la Torre^b^, Isabel Santacruz^b^, Oriol Vallcorba^a^, Monica Dapiaggi^c^, Susana G. Sanfélix^b,d^, and Miguel A.G. Aranda^a^*

^a^ ALBA Synchrotron, Carrer de la Llum 2-26. 08290 Cerdanyola del Vallès, Barcelona, Spain

^b^ Departamento de Química Inorgánica. Universidad de Málaga, 29071 Málaga, Spain

^c^ Department of Earth Sciences "Ardito Desio", University of Milan, Milano, Italy

^d^ Faculty of Engineering, Østfold University College, N-1757 Halden, Norway

* Correspondence: migarcia@cells.es or g_aranda@uma.es

*This supplementary information contains:*

**1. Extended Methods section.**

**2. Alite sample characterization.**

**3. Thermal analysis characterization for C_3_S_3μm_080_arrested:16d.**

**4. Supplementary tables:**

**Supplementary Table 1.** Selected results for the synchrotron PDF analysis for C_3_S_3μm_080_arrested:16d paste in the 10-25 Å r-region, using different structural descriptions for the nanocrystalline fraction of C-S-H phase.

**5. Supplementary Figures:**

**Supplementary Figure 1.** Particle size distribution (diameter) and cumulative measured in volume, of the following materials (a) as received alite, (b) vibratory milled alite and (c) attrition milled alite.

**Supplementary Figure 2.** SXRPD Rietveld plots at 14 hours of hydration for (a) C_3_S_21µm_080 and (b) C_3_S_7µm_080. The main peaks are labelled as follow: portlandite (•), alite () and added internal standard, SiO_2_ ().

**Supplementary Figure 3.** (a) LXRPD (Mo-Kα1 radiation) Rietveld plot for C_3_S_3μm_080_arrested:16d. The main peaks are labelled as follow: portlandite (•), alite () and added internal standard, α-Al_2_O_3_ (), (b) Simulated XRPD pattern for the defective clinotobermorite T3_14sc structure with particle size of approximately 5nm, using the same wavelength (Mo-Kα1 radiation) and (c) Raw SXRPD pattern for the PDF study of C_3_S_3μm_080_arrested:16d paste. SXRPD pattern for the empty capillary is also shown (red line).

**Supplementary Figure 4.** ^29^Si MAS-NMR spectra for (a) C3S_3µm_080_arrested:16d, (b) C3S_3µm_080_non-arrested:32d (a second preparation batch for the 3 μm alite sample) and (c) C3S_13µm_080_arrested:34d. The Mean Chain Length values are depicted and the intensity of the Q_0_ resonances indicates the unreacted alite fraction. Spinning rate of 15 kHz and a magnetic field of 14.1 T.

**Supplementary Figure 4.** High-angle annular dark-field scanning transmission electron (HAADF-STEM) micrograph for C_3_S_3μm_080_arrested:16d. Three independent analyses obtained by EDS are also included as examples.

**Supplementary Figure 5.** Field emission gun scanning electron (FEGSEM) micrograph for C_3_S_3μm_080_arrested:16d.

**Supplementary Figure 6.** Experimental (blue circles), fitted (red lines) and difference (grey lines) PDF patterns for C3S_3µm_080_arrested:16d from 10 to 25 Å using (a) clinotobermorite T3_14sc and (b) Jennite. The arrows highlight interatomic distance features poorly fitted by the Jennite structural description.

**Supplementary Figure 7.** Experimental (blue circles) and fitted (red solid line) PDF patterns for C_3_S_3μm_080_arrested:16d in the 2 to 15 Å r-range with three components: crystalline portlandite, unreacted alite and clinotobermorite. Difference curve is shown as a grey line which clearly shows the presence of an amorphous constituent. Simulated PDF curves for a monolayer (red) and a double layer (blue) calcium hydroxide, crystalline portlandite (green) and clinotobermorite T3_14sc (pink) are also included.

**Supplementary Figure 8.** Thermogravimetric data for C_3_S_3μm_080_arrested:16d.

**6. Description of every synchrotron powder diffraction raw data set deposited open access.**

**7. References.**

**1. Extended Methods section.**

**Sample description.** Monoclinic tricalcium silicate, alite, was acquired from Mineral Research Processing M.R.PRO. Its chemical composition determined by XRF was: 72.3 wt% CaO, 25.5 wt% SiO_2_, 1.1 wt% Fe_2_O_3_, 0.5 wt% MgO and 0.5 wt% Al_2_O_3_. For the *in situ* X-ray powder diffraction studies, the anhydrous mixtures were mixed with 10.00 wt% of SiO_2_ (99.5%, AlfaAesar) as an internal standard^1^. Pastes were prepared by mixing alite with water by hand in a small plastic baker for 1 min with a spatula and then immediately loaded into glass capillaries of 0.5 mm of diameter with a syringe. The capillaries were sealed with grease to avoid water loss. Capillaries which were prepared to be measured at ages of 7 days or later were kept inside closed plastic containers.

C_3_S_21µm_080 labels the paste produced by using as received alite with a water-to-alite mass ratio of 0.80. C_3_S_7µm_080: The as received alite was milled for 140 minutes, in cycles of 10 minutes with a rest-time between cycles of 10 minutes, in a vibratory mill (Retsch, mod. MM200). The resulting powder was mixed with a water-to-alite mass ratio of 0.80.

For the PDF study, as received alite was attrition milled (in house developed at UMA) with isopropanol for 4 hours in cycles of 10 minutes with a rest-time between cycles of 10 minutes and then dried at 100ºC for 1.5 hours. Then, this alite was hydrated at a water-to-solid mass ratio of 0.80 for 16 days. The paste was poured into a hermetically closed Teflon® cylinder for 1 day. Subsequently, the cylindrical paste was taken out and stored within demineralised boiled water for 16 days at 20ºC. Then, the paste was milled to fine powder in an agate mortar. Finally, to remove the excess of water, the sample was filtrated in a Whatman system (90 mm diameter Whatman filter with a pore size of 2.5 μm on a Teflon support) and washed twice with isopropanol and finally with ether^2^. This powder was filled in a glass capillary of 0.7 mm of diameter. This sample was labelled C_3_S_3 μm_080_arrested:16d.

**Particle Size Distribution (PSD).** Average particle size and particle size distribution for the alite samples were measured using a laser analyzer, Mastersizer S, Malvern, UK.

**Calorimetry.** The isothermal calorimetric study was performed in an eight channel Thermal Activity Monitor (TAM) instrument using glass ampoules. Pastes were prepared *ex situ* by mixing for 1 min ~3 g of each sample with the appropriated water, ~2 g, and they were immediately introduced in the calorimeter. A stabilization period of 45 minutes was needed to start the measurements. The heat flow was collected up to 7 days at 20ºC.

**Thermal analysis.** Differential thermal analysis (DTA) and thermogravimetric (TGA) measurement for C_3_S_3μm_080_arrested:16d was performed in a SDT-Q600 analyzer from TA instruments (New Castle, DE). The temperature was varied from RT to 1000°C at a heating rate of 10 °C/min. Measurements were carried out in open platinum crucibles under nitrogen flow.

**NMR study.** ^29^Si MAS-NMR (Magic Angle Spinning Nuclear Magnetic Resonance) spectrum for C_3_S_3μm_080_arrested:16d was recorded at RT on a Bruker AVIII HD 600 NMR spectrometer (field strength of 14.1 T) at 156.4 MHz with a 2.5 mm triple-resonance DVT probe using zirconia rotors at 15 kHz spinning rates. The experiment was performed with ^1^H decoupling (cw sequence) by applying a single pulse (π/2), an excitation pulse of 5 μs, 30 s relaxation delay and 10800 scans. The Chemical shift was referenced to an external solution of tetramethylsilane.

**Laboratory X-ray powder diffraction (LXRPD) with internal standard.** LXRPD data for C_3_S_3μm_080_arrested:16d was collected on a D8 ADVANCE (Bruker AXS) diffractometer (SCAI – Universidad de Malaga) equipped with a Johansson monochromator, using strictly monochromatic Mo-Kα_1_ radiation, λ=0.7093 Å, in transmission geometry (θ/θ). Samples were mixed with 20 wt% of α-Al_2_O_3_ (AlfaAesar 42571) as internal standard. α-Al_2_O_3_ was previously heated up to 1500°C for 20 hours and sieved <125 µm.

**Synchrotron X-ray powder diffraction (SXRPD).** For the phase evolution study, SXRPD patterns were collected in Debye-Scherrer (transmission) mode using the X-ray powder diffraction endstation of BL04-MSPD beamline at ALBA synchrotron (Barcelona, Spain)^3^. The wavelength, 0.61878(3) Å, was selected with a double-crystal Si (111) monochromator and determined by using Si640d NIST standard (a=5.43123 Å). The diffractometer is equipped with a MYTHEN detector especially suited for time-resolved and extremely good signal-to-noise ratio experiments. The glass capillaries, 0.5 mm of diameter, were rotated during data collection at a speed of 100 rpm to improve diffracting particle statistics. To improve the accuracy of the results, three SXRPD patterns were collect at three different positions of every capillary and merged to produce the final dataset. The total acquisition time was 6 min per dataset (2 minutes per pattern) over the angular range 1-35º (2θ). The temperature inside the experimental hutch was 28ºC.

For the PDF study, SXRPD data for C_3_S_3μm_080_arrested:16d were collected for 3 h at the same diffractometer. The employed wavelength was 0.41236(1) Å and the glass capillary diameter was 0.7 mm. Five patterns were collected, each lasted 37 min, and merged in order to improve the signal-to-noise ratio in the large recorded angular range, 1 to 120° (2θ). No changes between individual patterns were observed.

**Rietveld data analysis.** Rietveld analyses were performed using the GSAS suite of programs and the EXPGUI graphic interface^4^. Final global optimized parameters were: background coefficients, zero-shift error, cell parameters, and peak shape parameters using a pseudo-Voigt function. Portlandite and alite phases presented anisotropic lineshape broadening which was fitted by using the approach based of multidimensional distribution of lattice metrics^5^. For the portlandite crystal phase preferred orientation was also optimized by employing the March−Dollase ellipsoidal preferred orientation correction algorithm^6^. The non-crystalline content (amorphous and nanocrystalline) was determined by the internal standard methodology^7,8^.

**Pair Distribution Function data analysis.** PDF experimental data was obtained using PDFgetX3^9^ with Q_max_=21 Å^-1^. Quantitative phase analysis information was obtained from the PDF data by using the PDFgui software^10^. Final global optimized parameters were: scale factors, unit cell parameters and ADPs parameters. The delta2 value^11,12^ (low-r correlated motion peak sharpening factor) was fixed to 2 Å^2^. The instrumental parameters were obtained by measuring a similar data set for crystalline nickel. Nickel PDF data analysis converged to Qdamp=0.0030 Å^-1^ and Qbroad=0.0073 Å^-1^. The refinement of the difference curve (between 2 and 10 Å) was performed by means of CMI-diffpy complex modeling software^13^, with a structural model constituted of a nanoparticle of single-portlandite, with no assumption of periodicity. Final optimized parameters were: scale factor, stretching in the three different crystallographic directions, and ADP parameter for Ca atoms. This refined nanoparticle was then used (and the structural parameters fixed), together with the crystalline phases, in the fit of the PDF (2-10 Å) again with CMI-diffpy.

**Electron microscopy study.** High resolution transmission electron microscopy (HRTEM) measurements were carried out using a FEI Talos F200X microscope equipped with X FEG and super-X EDS system with four silicon drift detectors (SDDs) which operates at an accelerating voltage of 200 kV. Spectra were collected in Scanning TEM (STEM) mode. The sample was placed on a 200 mesh copper grid coated with formvar and carbon. For the field emission gun scanning electron microscopy (FEGSEM) study, the selected samples (ground powder) were covered with iridium. FEGSEM micrographs and EDS analysis were performed in a Helios Nanolab 650 Microscope (FEI Company) with a retractable CBS Backscatter detector (annular solid-state device) and X-Max 50 mm^2^ detector (Oxford instruments). Backscattering electron imaging (BSEI) was performed at 5 kV acceleration, and EDS analysis at 10 kV. The software AZtec (v.1.0) was used to quantify.

**2. Alite sample characterization.**

SXRPD data of the anhydrous as received alite, C_3_S_21µm, was analyzed by Rietveld methodology employing the internal standard method for amorphous quantification. A mixture between the M_1_ ^14^ and M_3_ ^15^ tricalcium silicate polymorphs were needed to obtain the best fit.

The Rietveld quantitative phase analysis gave the following phase assemblage: 38.7(3) wt% of M_1_-Ca_3_SiO_5_, 57.9(2) wt% of M_3_-Ca_3_SiO_5_ and 3.4(2) wt% of β-Ca_2_SiO_4_. The amorphous content was negligible for this sample. The final unit cell parameters for the M_1_-Ca_3_SiO_5_ converged to a=9.2990(2) Å, b=7.0847(1) Å, c=12.1984(3) Å and β=116.144(1)° and for the M_3_-Ca_3_SiO_5_ were a=33.1289(8) Å, b=7.0561(2) Å, c=18.5844(4) Å and β=94.247(2)°.

The same analysis was performed for the vibratory milled sample, C_3_S_7µm. The quantitative phase analysis results obtained were the following: 31.6(3) wt% of M_1_-Ca_3_SiO_5_, 62.6(2) wt% of M_3_-Ca_3_SiO_5_, 2.6(1) wt% of β-Ca_2_SiO_4_ and 3.2(1) wt% of amorphous content. The final unit cell parameters for the M_1_-Ca_3_SiO_5_ were a=9.2999(2) Å, b=7.0857(2) Å, c=12.1995(3) Å and β=116.148(2)° and for the M_3_-Ca_3_SiO_5_ were a=33.1302(9) Å, b=7.0575(3) Å, c=18.5840(5) Å and β=94.32(2)°.

The Rietveld analysis for the attrition milled sample, C_3_S_3 μm, was performed by using LXRPD data. The Rietveld quantitative phase analysis was the following: 26.8(8) wt% of M_1_-Ca_3_SiO_5_, 44.6(9) wt% of M_3_-Ca_3_SiO_5_, 2.4(2) wt% of β-Ca_2_SiO_4_ and 26.3(1) wt% of amorphous content. The final unit cell parameters for the M_1_-Ca_3_SiO_5_ converged to a=9.2900(8) Å, b=7.0714(11) Å, c=12.1878(17) Å and β=116.157(8)° and for the M_3_-Ca_3_SiO_5_ were a=33.051(17) Å, b=7.066(2) Å, c=18.526(4) Å and β=94.42(4)°.

Finally, The PDF pattern of as received Ca_3_SiO_5_ was fitted with the M3 structure of Mumme ^16^ by using PDFGui. The obtained unit cell parameters and atomic displacement parameters (ADPs) were: a=12.213 Å, b=7.085 Å, c=9.304 Å and β=116.1°; and 0.018, 0.011 and 0.070 Å^2^ for Ca, Si and O, respectively. These values were used for the analysis of the minor fraction present in the sample C3S_3μm_080_arrested:16d paste and only the scale factor for alite was refined.

**3. Thermal analysis characterization for C_3_S_3μm_080_arrested:16d.**

The thermogravimetric curve for C_3_S_3μm_080_arrested:16d paste is shown in Supplementary Fig. 8. Several mass loss stages are observed in thermal trace. The mass loss from RT to 250°C, 17.7 wt%, is mainly ascribed to the water release from C-S-H gel aggregates. The weight loss measured between 250°C and 400°C, 2.4 wt%, could be partly related with the dehydration of amorphous calcium hydroxide. 5.7 wt% mass loss is measured between 400°C and 550°C, centered at 455°C, which corresponds to the water loss from crystalline portlandite. Two final weight losses are evident from 550 to 1000 ºC. The one observed close to 755ºC, ≈2.0 wt%, is likely due to the CO_2_ released of crystalline calcite. The other close to 660°C, ≈3.8 wt%, is very likely related to the CO_2_ release from amorphous calcium carbonate and probably also to the release of organic solvent, used for the arresting of the hydration, absorbed in the C-S-H gel as previously reported^17^.

The components of the gel are nanocrystalline defective clinotobermorite, amorphous (monolayer) calcium hydroxide, and water within the nanopores. The weight losses for each component can be compared with that experimentally obtained for the C3S_3μm_080_arrested:16d paste. For full reaction, the theoretical weight loss for the water gel pores, 13.27 wt%, plus the water in clinotorbemorite, 5.28 wt%, sum 18.55 wt% which is comparable with the experimental weight loss between RT to 250ºC, 17.7 wt% for this paste. Secondly, the calculated weight loss from Si-OH condensation and from amorphous Ca(OH)_2_ is 0.62 and 3.24 wt%, respectively which can be compared with the water loss observed between 250°C and 400°C, 2.4 wt% plus 2.1 wt% from the contribution of the carbonation. Finally, the experimental water loss from crystalline portlandite, 5.7 wt% is compatible with the calculated value according to reaction (1), main text, 6.7 wt%.

**4. Supplementary tables:**

**Supplementary Table 1.** Selected results for the synchrotron PDF analysis for C_3_S_3μm_080_arrested:16d paste in the 10-25 Å r-region, using different structural descriptions for the nanocrystalline fraction of C-S-H gel.

| **Phase** | **R_W_ (%)** | **Ca_3_SiO_5_ (wt%)** | **Crystalline Ca(OH)_2_ (wt%)** | **Nanocrystalline fraction of C-S-H (wt%)** |
| --- | --- | --- | --- | --- |
| Hillebrandite (o), ICSD #80127 | 33.4 | 3.7 | 55.6 | 40.7 |
| Jennite (t), ICSD #151413 | 33.5 | 3.3 | 46.5 | 50.2 |
| Tobermorite-14 (m), ICSD #152489 | 33.1 | 2.9 | 44.5 | 52.5 |
| Tobermorite-11 (o), ICSD #92941 | 28.5 | 2.5 | 38.3 | 59.1 |
| Tobermorite-11 (m), ICSD #87690 | 28.7 | 2.4 | 36.9 | 60.7 |
| Tobermorite-11 (o), ICSD #100405 | 28.4 | 2.5 | 37.2 | 60.4 |
| Clinotobermorite (m), ICSD #90036 | 27.4 | 1.8 | 27.1 | 71.1 |
| Clinotobermorite (t), ICSD #90034 | 27.9 | 2.0 | 29.8 | 68.3 |
| Clinotobermorite (m), T5_14sc* | 28.3 | 2.1 | 32.3 | 65.5 |
| Clinotobermorite (m), T5_11sc* | 28.4 | 1.9 | 28.8 | 69.3 |
| **Clinotobermorite (m), T3_14sc*** | **27.7** | **2.2** | **33.5** | **64.3** |

*Richardson, 2014 ^18^; (m), (o) & (t) denotes monoclinic, orthorhombic and triclinic, respectively.

**5. Supplementary Figures:**

**Supplementary Figure 1.** Particle size distribution (diameter) and cumulative measured in volume, of the following materials (a) as received alite, (b) vibratory milled alite and (c) attrition milled alite.

**Supplementary Figure 2.** SXRPD Rietveld plots at 14 hours of hydration for (a) C_3_S_21µm_080 and (b) C_3_S_7µm_080. The main peaks are labelled as follow: portlandite (•), alite () and added internal standard, SiO_2_ ().

**Supplementary Figure 3.** (a) LXRPD (Mo-Kα1 radiation) Rietveld plot for C_3_S_3μm_080_arrested:16d. The main peaks are labelled as follow: portlandite (•), alite () and added internal standard, α-Al_2_O_3_ (), (b) Simulated XRD pattern for the defective clinotobermorite T3_14sc structure with particle size of approximately 5nm, using the same wavelength (Mo-Kα1 radiation) and (c) Raw SXRPD pattern for the PDF study of C_3_S_3μm_080_arrested:16d paste. SXRPD pattern for the empty capillary is also shown (red line)

**Supplementary Figure 4.** ^29^Si MAS-NMR spectra for (a) C3S_3µm_080_arrested:16d, (b) C3S_3µm_080_non-arrested:32d (a second preparation batch for the 3 μm alite sample) and (c) C3S_13µm_080_arrested:34d. The Mean Chain Length values are depicted and the intensity of the Q_0_ resonances indicates the unreacted alite fraction. Spinning rate of 15 kHz and a magnetic field of 14.1 T.

**
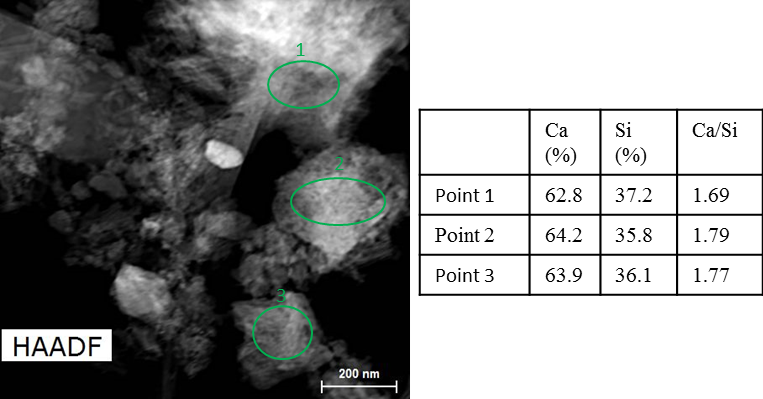
**

**Supplementary Figure 5.** High-angle annular dark-field scanning transmission electron (HAADF-STEM) micrograph for C_3_S_3μm_080_arrested:16d. Three independent analyses obtained by EDS are also included as examples.

**Supplementary Figure 6.** Field emission gun scanning electron (FEGSEM) micrograph for C_3_S_3μm_080_arrested:16d.

**Supplementary Figure 7.** Experimental (blue circles), fitted (red lines) and difference (grey lines) PDF patterns for C3S_3µm_080_arrested:16d from 10 to 25 Å using (a) clinotobermorite T3_14sc and (b) Jennite. The arrows highlight interatomic distance features poorly fitted by the Jennite structural description.

**Supplementary Figure 8.** Experimental (blue circles) and fitted (red solid line) PDF patterns for C_3_S_3μm_080_arrested:16d in the 2 to 15 Å r-range with three components: crystalline portlandite, unreacted alite and clinotobermorite. Difference curve is shown as a grey line which clearly shows the presence of an amorphous constituent. Simulated PDF curves for a monolayer (red) and a double layer (blue) calcium hydroxide, crystalline portlandite (green) and clinotobermorite T3_14sc (pink) are also included.

**Supplementary Figure 9.** Thermogravimetric data for C_3_S_3μm_080_arrested:16d.

**6. Description of every synchrotron powder diffraction raw data set deposited open access.**

All synchrotron X-ray powder diffraction raw data files underlying this article can be accessed on Zenodo at https://doi.org/10.5281/zenodo.1027759, and used under the Creative Commons Attribution license.

**Files:**

**Synchrotron X-ray powder diffraction study for quantitative analysis**

C3S_anh_ALL.dat: anhydrous C_3_S_21µm

C3S_080_cap1_5h_ALL.dat: C_3_S_21µm_080 at 5h of hydration

C3S_080_cap1_ALL_01_1707132228.dat: C_3_S_21µm_080 at 5.5h of hydration

C3S_080_cap1_ALL_02_1707132300.dat: C_3_S_21µm_080 at 6h of hydration

C3S_080_cap1_ALL_03_1707132331.dat: C_3_S_21µm_080 at 6.5h of hydration

C3S_080_cap1_ALL_04_1707140003.dat: C_3_S_21µm_080 at 7h of hydration

C3S_080_cap1_ALL_05_1707140034.dat: C_3_S_21µm_080 at 7.5h of hydration

C3S_080_cap1_ALL_06_1707140105.dat: C_3_S_21µm_080 at 8h of hydration

C3S_080_cap1_ALL_07_1707140137.dat: C_3_S_21µm_080 at 8.75h of hydration

C3S_080_cap1_ALL_08_1707140208.dat: C_3_S_21µm_080 at 9.25h of hydration

C3S_080_cap1_ALL_09_1707140239.dat: C_3_S_21µm_080 at 9.75h of hydration

C3S_080_cap1_ALL_10_1707140311.dat: C_3_S_21µm_080 at 10.25h of hydration

C3S_080_cap1_ALL_11_1707140342.dat: C_3_S_21µm_080 at 10.75h of hydration

C3S_080_cap1_ALL_12_1707140414.dat: C_3_S_21µm_080 at 11.25h of hydration

C3S_080_cap1_ALL_13_1707140445.dat: C_3_S_21µm_080 at 11.75h of hydration

C3S_080_cap1_ALL_14_1707140516.dat: C_3_S_21µm_080 at 12.25h of hydration

C3S_080_cap1_ALL_15_1707140548.dat: C_3_S_21µm_080 at 13h of hydration

C3S_080_cap1_ALL_16_1707140619.dat: C_3_S_21µm_080 at 13.5h of hydration

C3S_080_cap1_ALL_17_1707140650.dat: C_3_S_21µm_080 at 14h of hydration

C3S_080_cap1_ALL_18_1707140722.dat: C_3_S_21µm_080 at 14.5h of hydration

C3S_080_cap1_ALL_19_1707140753.dat: C_3_S_21µm_080 at 15h of hydration

C3S_080_cap1_ALL_20_1707140825.dat: C_3_S_21µm_080 at 15.5h of hydration

C3S_080_cap1_17h_ALL.dat: C_3_S_21µm_080 at 17.5h of hydration

C3S_080_cap1_22h_ALL.dat: C_3_S_21µm_080 at 22h of hydration

C3S_080_cap1_24h_ALL.dat: C_3_S_21µm_080 at 24h of hydration

C3S_080_cap1_28h_ALL.dat: C_3_S_21µm_080 at 27.5h of hydration

C3S_080_cap2_36h_ALL.dat: C_3_S_21µm_080 at 36h of hydration

C3S_080_cap2_39h_ALL.dat: C_3_S_21µm_080 at 39h of hydration

C3S_080_cap1_42h_ALL.dat: C_3_S_21µm_080 at 42h of hydration

C3S_080_cap2_44h_ALL.dat: C_3_S_21µm_080 at 44h of hydration

C3S_080_cap1_48h_ALL.dat: C_3_S_21µm_080 at 48h of hydration

C3S_080_cap1_52h_ALL.dat: C_3_S_21µm_080 at 52h of hydration

C3S_080_7d_ALL.dat: C_3_S_21µm_080 at 7d of hydration

C3S_080_14d_rep_ALL.dat: C_3_S_21µm_080 at 14d of hydration

C3S_080_28d_ALL.dat: C_3_S_21µm_080 at 28d of hydration

C3S_080_3m_ALL.dat: C_3_S_21µm_080 at 3m of hydration

C3Sm_anh_ALL.dat: anhydrous C_3_S_7µm_080

C3Sm_080_cap3_2h_ALL.dat: C_3_S_7µm_080 at 2h of hydration

C3Sm_080_cap3_4h_ALL.dat: C_3_S_7µm_080 at 4h of hydration

C3Sm_080_cap1_ALL_01_1707132243.dat: C_3_S_7µm_080 at 5.75h of hydration

C3Sm_080_cap1_ALL_02_1707132315.dat: C_3_S_7µm_080 at 6.25h of hydration

C3Sm_080_cap1_ALL_03_1707132346.dat: C_3_S_7µm_080 at 6.75h of hydration

C3Sm_080_cap1_ALL_04_1707140018.dat: C_3_S_7µm_080 at 7.25h of hydration

C3Sm_080_cap1_ALL_05_1707140049.dat: C_3_S_7µm_080 at 7.75h of hydration

C3Sm_080_cap1_ALL_06_1707140120.dat: C_3_S_7µm_080 at 8.25h of hydration

C3Sm_080_cap1_ALL_07_1707140152.dat: C_3_S_7µm_080 at 8.45h of hydration

C3Sm_080_cap1_ALL_08_1707140223.dat: C_3_S_7µm_080 at 9.25h of hydration

C3Sm_080_cap1_ALL_09_1707140254.dat: C_3_S_7µm_080 at 10h of hydration

C3Sm_080_cap1_ALL_10_1707140326.dat: C_3_S_7µm_080 at 10.5h of hydration

C3Sm_080_cap1_ALL_11_1707140357.dat: C_3_S_7µm_080 at 11h of hydration

C3Sm_080_cap1_ALL_12_1707140429.dat: C_3_S_7µm_080 at 11.5h of hydration

C3Sm_080_cap1_ALL_13_1707140500.dat: C_3_S_7µm_080 at 12h of hydration

C3Sm_080_cap1_ALL_14_1707140531.dat: C_3_S_7µm_080 at 12.5h of hydration

C3Sm_080_cap1_ALL_15_1707140603.dat: C_3_S_7µm_080 at 13h of hydration

C3Sm_080_cap1_ALL_16_1707140634.dat: C_3_S_7µm_080 at 13.5h of hydration

C3Sm_080_cap1_ALL_17_1707140706.dat: C_3_S_7µm_080 at 14h of hydration

C3Sm_080_cap1_ALL_18_1707140737.dat: C_3_S_7µm_080 at 14.5h of hydration

C3Sm_080_cap1_ALL_19_1707140808.dat: C_3_S_7µm_080 at 15h of hydration

C3Sm_080_cap1_ALL_20_1707140840.dat: C_3_S_7µm_080 at 15.5h of hydration

C3Sm_080_cap1_18h_ALL.dat: C_3_S_7µm_080 at 18h of hydration

C3Sm_080_cap1_20h_ALL.dat: C_3_S_7µm_080 at 20h of hydration

C3Sm_080_cap1_24h_ALL.dat: C_3_S_7µm_080 at 24h of hydration

C3Sm_080_cap1_28h_ALL.dat: C_3_S_7µm_080 at 28h of hydration

C3Sm_080_cap2_35h_ALL.dat: C_3_S_7µm_080 at 35.5h of hydration

C3Sm_080_cap2_39h_ALL.dat: C_3_S_7µm_080 at 38.5h of hydration

C3Sm_080_cap1_42h_ALL.dat: C_3_S_7µm_080 at 42.5h of hydration

C3Sm_080_cap2_44h_ALL.dat: C_3_S_7µm_080 at 44h of hydration

C3Sm_080_cap1_48h_ALL.dat: C_3_S_7µm_080 at 48h of hydration

C3Sm_080_cap1_52h_ALL.dat: C_3_S_7µm_080 at 52h of hydration

C3Sm_080_7d_ALL.dat: C_3_S_7µm_080 at 7d of hydration

C3Sm_080_14d_ALL.dat: C_3_S_7µm_080 at 14d of hydration

C3Sm_080_28d_b_ALL.dat: C_3_S_7µm_080 at 28d of hydration

C3S_milled_080_3m_ALL.dat: C_3_S_7µm_080 at 3m of hydration

**Pair Distribution Function study for nanoscale analysis and characterization**

empty0p7_ALL.dat: empty capillary.

Ni0p7_ALL.dat: Nickel sample employed as standard.

C3S_080_milled_Marzo_ALL.dat: C_3_S_3μm_080_arrested:16d.

**7. References**

1. Cuesta, A. *et al.* Hydration mechanisms of two polymorphs of synthetic ye’elimite. *Cem. Concr. Res.* **63,** 127–136 (2014).

2. García-Mate, M., De la Torre, A. G., Leon-Reina, L., Aranda, M. A. G. & Santacruz, I. Hydration studies of calcium sulfoaluminate cements blended with fly ash. *Cem. Concr. Res.* **54,** 12−20 (2013).

3. Fauth, F., Peral, I., Popescu, C. & Knapp, M. The new Material Science Powder Diffraction beamline at ALBA Synchrotron. *Powder Diffr.* **28,** S360−S370 (2013).

4. Larson, A. C. & Von Dreele, R. B. *General Structure Analysis System (GSAS).* Los Alamos National Laboratory Report LAUR, pp 86−748 (2000).

5. Stephens, P.W. Phenomenological model of anisotropic peak broadening in powder diffraction. *J. Appl. Crystallogr.* **32,** 281−289 (1999).

6. Dollase, W.A. Correction of intensities for preferred orientation in powder diffractometry: application of the March model. *J. Appl. Crystallogr.* **19,** 267−272 (1986).

7. Aranda, M. A. G., De la Torre, A. G. & León-Reina, L. Rietveld quantitative phase analysis of OPC clinkers, cements and hydration products. *Rev. Mineral. Geochem.* **74,** 169−209 (2012).

8. De la Torre, A. G., Bruque, S. & Aranda, M. A. G. Rietveld quantitative amorphous content analysis. *J. Appl. Crystallogr.* **34,** 196−202 (2001).

9. Juhàs, P., Davis, T., Farrow, C.L. & Billinge, S. J. L. PDFgetX3: a rapid and highly automatable program for processing powder diffraction data into total scattering pair distribution functions. *J. Appl. Crystallogr.* **46,** 560−566 (2013).

10. Farrow, C.L. *et al.* PDFfit2 and PDFgui: computer programs for studying nanostructure in crystals. *J. Phys. Condens. Matter.* **19,** 335219 (2007).

11. Jeong, I.-K., Heffner, R.H., Graf, M.J. & Billinge, S. J. L. Lattice dynamics and correlated atomic motion from the atomic pair distribution function. *Phys. Rev. B* **67,** 104301 (2003).

12. Jeong, I.-K., Proffen, T., Mohiuddin-Jacobs, F. & Billinge, S.J.L. Measuring Correlated Atomic Motion Using X-ray Diffraction. *J. Phys. Chem. A* **103,** 921−924 (1999).

13. Juhás, P., Farrow, C.L., Yang, X., Knox, K. R. & Billing, S. J. L. Complex modeling: a strategy and software program for combining multiple information sources to solve ill posed structure and nanostructure inverse problems. *Acta Crystallogr. A* **71,** 562−568 (2015).

14. De Noirfontaine, M. N., Dunstetter, F., Courtial, M., Gasecki, G. & Signes-Frehel, M. Polymorphism of tricalcium silicate, the major compound of Portland cement clinker: 2. Modelling alite for Rietveld analysis, an industrial challenge. *Cem Concr Res* **36,** 54–64 (2006).

15. Nishi, F., Takeuchi, Y. & Maki, I. Tricalcium silicate Ca_3_O[SiO_4_]: The Monoclinic superstructure. *Z. Kristallogr.* **172,** 297−314 (1985).

16. Mumme, W.G. Crystal structure of tricalcium silicate from a Portland cement clinker and its application to quantitative XRD analysis. *N. Jb. Miner. Abh.* **4,** 145–160 (1995).

17. Zhang, J. & Scherer, W. Comparison of methods for arresting hydration of cements. *Cem. Concr. Res*. **41,** 1024−1036 (2011).

18. Richardson, I.G. Model structures for C-(A)-S-H(I). *Acta Crystallogr. B* **70,** 903−923 (2014).
